# An Improved Method for the Extraction of Nucleic Acids from Plant Tissue without Grinding to Detect Plant Viruses and Viroids

**DOI:** 10.3390/plants10122683

**Published:** 2021-12-06

**Authors:** Tatsuji Hataya

**Affiliations:** Pathogen-Plant Interactions, Research Faculty of Agriculture, Hokkaido University, Kita 9, Nishi 9, Kita-ku, Sapporo 060-8589, Japan; hataya@res.agr.hokudai.ac.jp

**Keywords:** molecular diagnostic, diagnosis, RT-PCR, xanthogenate, internal control RNA

## Abstract

Gene amplification techniques such as polymerase chain reaction (PCR) are widely used for the diagnosis of plant diseases caused by viruses and viroids. It is preferable that sample preparation methods for PCR or reverse transcription (RT) PCR are rapid, straightforward, and inexpensive. We previously reported a method for the extraction of nucleic acids without mechanical tissue grinding using a buffer containing potassium ethyl xanthogenate (PEX) to detect viroid RNAs. In the present report, the previous PEX method was improved and simplified. In the simplified PEX (SPEX) method, the process of PEX buffer treatment for plant cell wall disruption is improved to one step of incubation at 80 °C for 10 min, instead of three steps that took more than 26 min at 65 °C in the previous method. Total nucleic acids could be extracted from fresh, frozen, or dried leaves of a cultivar or wild species of tobacco, tomato, citron, hop plants, and pericarps of persimmon fruits by the SPEX method. Several RNA viruses and viroids were successfully detected from the extracted nucleic acids together with an internal mRNA by RT-PCR. The SPEX method may be useful for detecting not only viruses and viroids, but also other plant pathogens.

## 1. Introduction

Plant viruses and viroids are responsible for substantial losses in yield and reduce the quality of fruits and flowers in many crops worldwide [1]. Early diagnosis of viral and viroid diseases is critical for disease control because there are no substantially effective agricultural chemicals that act directly on these pathogens. Polymerase chain reaction (PCR) and reverse transcription (RT) PCR are useful for detecting plant viruses, especially some viruses that are difficult to apply serological detection methods. Unlike viruses, viroids are circular, single-stranded RNA molecules known as the smallest plant pathogens and do not encode any proteins [2]; thus, they cannot be detected by serological methods [3]. Consequently, RT-PCR is widely used for detecting viroids in addition to some plant viruses not only for disease diagnosis, but also in laboratory studies.

However, the application of highly sensitive PCR for diagnostics by the amplification of various target sequences is often hampered by problems of false-positives generated with nucleic acid contamination, e.g., (i) cross-contamination among samples, (ii) carry-over contamination from previous amplified products, (iii) laboratory workstation surface contamination, (iv) contamination of laboratory instruments and equipment including micropipettes, tube racks, centrifuge, vortex mixer, (v) contamination in the PCR reagents. Effective decontamination procedures for the aforementioned contaminations (ii)–(v) have been reported, including ultraviolet (UV) irradiation and hypochlorous acid solution, which can effectively eliminate nucleic acids on the work surface, laboratory instruments, and equipment. The degradation by uracil-N-glucosidase of PCR products incorporating dUTP instead of dTTP during PCR is efficient for the elimination of carry-over contamination [4,5,6,7,8]. In contrast, cross-contamination among samples is difficult to prevent completely. Protocols of PCR and RT-PCR in plant disease diagnosis usually uses nucleic acids extracted from plant tissue as a template, and therefore cross-contamination occurs easily in the process of nucleic acid extraction. Viroids and most plant virus genomes are RNA, and various methods have been developed for the extraction of total RNA from plant tissues. Various commercial kits are also available, although these kits are expensive to use for routine plant disease diagnosis and especially for a large number of samples. Many methods for plant RNA extraction include tissue grinding, which leads to a higher risk of cross-contamination, especially at the time of manual grinding using a mortar and pestle. The risk of cross-contamination increases as the extraction process proceeds. Therefore, a simple process using limited and disposable equipment is desired for the extraction procedure in routine of plant disease diagnosis and laboratory studies.

Jhingan [9] developed a simple method to extract plant genomic DNA using potassium ethyl xanthogenate (PEX), which damages plant cell walls, subsequently disrupting cells and releasing the DNA without grinding tissues and removing proteins. DNA was extracted from 1 g of fresh leaf tissue by incubation at 65 °C for 20 min in 4 mL of PEX buffer in a 15 mL tube. Williams and Ronald [10] modified the protocol of Jhingan to small-scale using a microcentrifuge tube. Frozen leaf tissue of 1/3 cm^2^ was dipped in 70% ethanol prior to placing it in 0.1 mL of PEX buffer so as to be fully submerged. In addition, plant cell wall disruption with the PEX buffer was performed in three steps, consisting of an incubation at 65 °C for 5 min, the infiltration of the PEX buffer into plant tissues in a microcentrifuge tube with an opened cap using a centrifuge evaporator, and subsequent incubation at 65 °C for 15 min. We applied the protocol of Williams and Ronald [10] for the first time to the extraction of viroid RNA from 0.1–0.3 g of host plant tissues [11]. However, the occurrence of cross-contamination in the infiltration step of the PEX buffer into plant tissues was of concern due to the use of cap-opened tube. In order to decrease cross-contamination in this process as much as possible in theory, we improved in this study the previous standard PEX method based on Williams and Ronald [10] to one step of PEX buffer treatment instead of three steps. Herein, we report a simplified PEX (SPEX) method for the detection of not only viroids, but also plant viruses in combination with a plant mRNA internal control that is useful to evaluate the quality of extracted nucleic acids sufficient for RT-PCR.

## 2. Results

### 2.1. Incubation Conditions of Leaf Discs with PEX Buffer

Leaf tissue of 0.1–0.3 g was used in the previous PEX method [11], whereas leaf discs with a reduced amount of 0.05–0.1 g was used in the modified SPEX method so as to easily replicate the amount of tissue from each test plant by the number of discs, to easily transfer plant tissue cut out from leaf to the centrifuge tube using a plastic straw, and to fully immerse the tissue in the PEX buffer for plant cell wall disruption efficiently. In addition, the modified SPEX method was conducted by one step of incubation in the same way as Jhingan [9], but it was shortened to 10 min at various temperatures.

**Figure 1:**
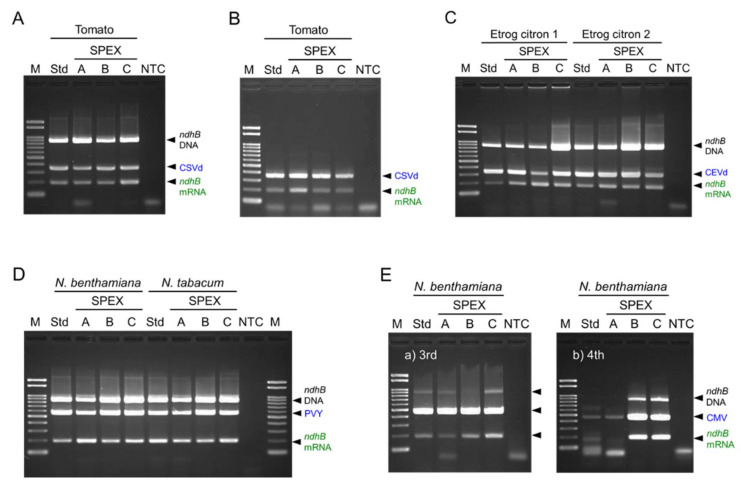
Comparison of incubation conditions in the SPEX method. Chrysanthemum stunt viroid (CSVd) in tomato (**A**,**B**), citrus exocortis viroid (CEVd) in ‘Etrog’ citron (**C**), potato virus Y (PVY) in *Nicotiana benthamiana* and *N*. *tabacum* (**D**), or cucumber mosaic virus (CMV) in *N*. *benthamiana* (**E**, the 3rd and 4th experiments) were simultaneously detected in combination with NADH dehydrogenase subunit 2 mRNA (*ndhB*) by RT-PCR using Go-to DNA polymerase (**A**,**C**–**E**) or Hot Start *Taq* DNA polymerase (**B**). Nucleic acids were extracted from leaves using the standard PEX (lane Std)*,* and SPEX-A, SPEX-B, and SPEX-C (lanes **A**–**C**) methods. The identical cDNA products were used in (**A**) and (**B**). Duplex RT-PCR products were electrophoresed in a 2% agarose gel. Lanes, NTC: no template control; M: molecular size marker Gene Ladder 100.

Nucleic acids were extracted from leaves of tomato infected with chrysanthemum stunt viroid (CSVd, genus *Pospiviroid*) by incubation for 10 min at 65 °C (SPEX-A), 80 °C (SPEX-B), or 95 °C (SPEX-C), in addition to the standard PEX procedure. Both cDNA fragments of CSVd and plant mRNA could be amplified from all nucleic acid extracts (Figure 1A). Although the forward primer AtropaNad2.1a was designed to be specific to mRNA of NADH dehydrogenase subunit 2 (*ndhB* gene) by spanning the splice junction [12], an 867 bp fragment containing an intron sequence of 679 bp derived from the genomic *ndhB* DNA sequence was amplified by mispriming, especially in the presence of large amounts of genomic DNA, as previously shown by the disappearance of the band upon DNase I treatment before RT-PCR [13]. This non-specific amplified product of *ndhB* DNA appeared in large amounts when using Go-to DNA polymerase (Figure 1A), but less when using Hot Start *Taq* DNA polymerase (Figure 1B). However, appearance of both amplified products derived from *ndhB* genomic DNA and mRNA indicates that both DNA and RNA were extracted; therefore, Go-to DNA polymerase was used in this study owing to the evaluation of extracted DNA and RNA.

Similar amplification was observed in all RT-PCR products for the simultaneous detection of citrus exocortis viroid (CEVd, genus *Pospiviroid*) and *ndhB* mRNA using nucleic acids extracted from the young, soft, and pliable leaves of ‘Etrog’ citron (*Citrus medica*) (Figure 1C). In addition, potato virus Y (PVY, genus *Potyvirus*) and *ndhB* mRNA were successfully detected in all nucleic acid extracts of *Nicotiana benthamiana* plants 14 days post-inoculation (dpi), and tobacco (*N. tabacum*) plants 10 dpi (Figure 1D). When cucumber mosaic virus (CMV, genus *Cucumovirus*) and *ndhB* mRNA were tested simultaneously from nucleic acid extracts of *N. benthamiana* 10 or 14 dpi, the duplex RT-PCR sometimes resulted in no or poor amplification from nucleic acids extracted using the standard PEX and SPEX-A methods (Figure 1(Eb)) although both targets were sometimes successfully detected in all nucleic acid extracts (Figure 1(Ea)). Besides these two results, the other three results are shown in Figure S1 and the results of five repetitive experiments in total are summarized in Table 1. These results suggest that the nucleic acid extraction using the SPEX-B and SPEX-C methods may be more stable for the detection of target RNA than that using the standard PEX and SPEX-A methods by incubation at 65 °C.

### 2.2. Quality and Quantity of Extracted Nucleic Acids

**Figure 2:**
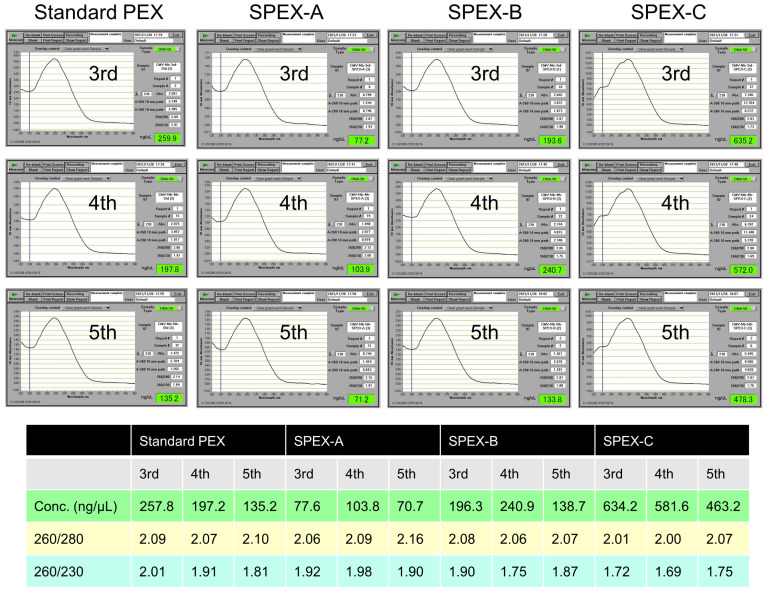
Evaluation of total nucleic acids extracted by the standard PEX and three SPEX methods. The 3rd, 4th, and 5th nucleic acid extracts of *N. benthamiana* infected with CMV, which were used in experiments of Table 1, were evaluated using a NanoDrop 1000 spectrophotometer. Each UV-absorbance spectrum is the one showing the median nucleic acid concentration among three measurements. The mean value of nucleic acid concentration and absorbance ratio was calculated.

The quality and quantity of total nucleic acids of *N. benthamiana* leaves extracted by the standard PEX and three SPEX methods were assessed using a NanoDrop™ 1000 spectrophotometer. The aforementioned 3rd, 4th, and 5th nucleic acid extracts of *N. benthamiana* infected with CMV (Figure 1E and Figure S1, Table 1) were used for the assessment. Absorbance value was measured three times per nucleic acid extract. In addition to each UV-absorbance spectrum showing the median nucleic acid concentration among three measurements, each calculated mean value of nucleic acid concentration and absorbance ratio is presented in Figure 2. The concentration of nucleic acids extracted by the standard PEX method ranged from 135.2–257.8 ng/µL and was similar to 138.7–240.9 ng/µL by the SPEX-B method. The concentration of nucleic acids was the lowest in 70.7–103.8 ng/µL by the SPEX-A method and the highest in 463.2–634.2 ng/µL by the SPEX-C method. The absorbance ratio of 260/280 nm ranged from 2.00–2.16 in all nucleic acid extracts, which indicates relatively pure nucleic acids. Meanwhile, the absorbance ratio of 260/230 nm lower than 1.8 was observed in the 4th extract by the SPEX-B method and all three extracts by the SPEX-C method, suggesting relatively impure nucleic acids [14]. Therefore, the extraction by the SPEX-C method provided the larger quantity, but lower quality of total nucleic acids than the three other methods. Consistent with the presence of impurity in total nucleic acids extracted by the SPEX-C method, the pellet containing nucleic acids precipitated with 2-propanol tended to be larger than that by other methods and to be difficult to dissolve in 20 µL of ultrapure water.

### 2.3. Nucleic Acid Extraction from Preserved Leaves Using the SPEX Method

To examine the possibility of SPEX method application for the detection of plant viruses and viroids from preserved leaves, CSVd-infected tomato and CMV-infected *N. benthamiana* leaves were frozen at −80 °C or −20 °C, or dried using silica gel in a refrigerator for 63 and 61 days, respectively. For comparison, nucleic acids extracted from fresh leaves were stored at −80 °C until RT-PCR analysis.

**Figure 3:**
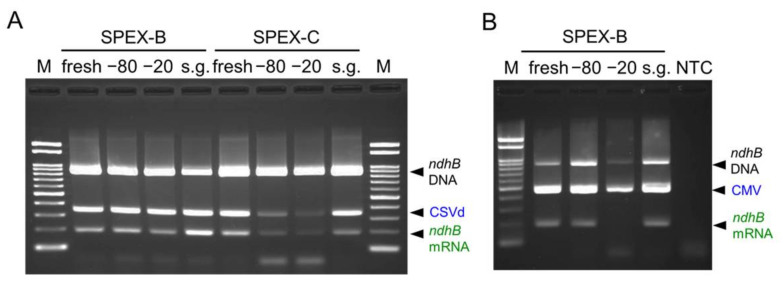
Comparison of leaf preserving conditions for use in the SPEX method. Chrysanthemum stunt viroid (CSVd) in tomato (**A**) or cucumber mosaic virus (CMV) in *N. benthamiana* (**B**) was simultaneously detected in combination with *ndhB* mRNA using nucleic acids extracted from fresh or preserved leaves by the SPEX-B and SPEX-C methods. Preserving conditions were compared by freezing at −80 °C (lane −80) and −20 °C (lane −20) and drying using silica gel (lane s.g.). Duplex RT-PCR products were electrophoresed in a 2% agarose gel. Lanes, NTC: no template control; M: molecular size marker Gene Ladder 100.

In the SPEX-B method, both cDNA fragments of CSVd and *ndhB* mRNA could be amplified from frozen leaves at −80 °C and −20 °C, and silica gel-dried leaves as well as fresh leaves (Figure 3A). Meanwhile, in the SPEX-C method, the cDNA product of CSVd and *ndhB* mRNA was poorly amplified from frozen leaves at −80 °C and −20 °C, although the genomic *ndhB* DNA sequence was amplified from all nucleic acid extracts (Figure 3A). Using the SPEX-B method, CMV and *ndhB* mRNA could be detected from −80 °C frozen leaves and silica gel-dried leaves as well as fresh leaves of *N. benthamiana*; however, a lower amount of the CMV band and no *ndhB* mRNA band was observed in RT-PCR products amplified from nucleic acid extracts of −20 °C frozen leaves (Figure 3B). These results indicate that the SPEX-B method is applicable to −80 °C frozen and silica gel-dried leaves as well as from fresh leaves for the reliable detection of *ndhB* mRNA, CSVd, and CMV.

### 2.4. Application of the SPEX Method for the Detection of Pathogens from Crops

**Figure 4:**
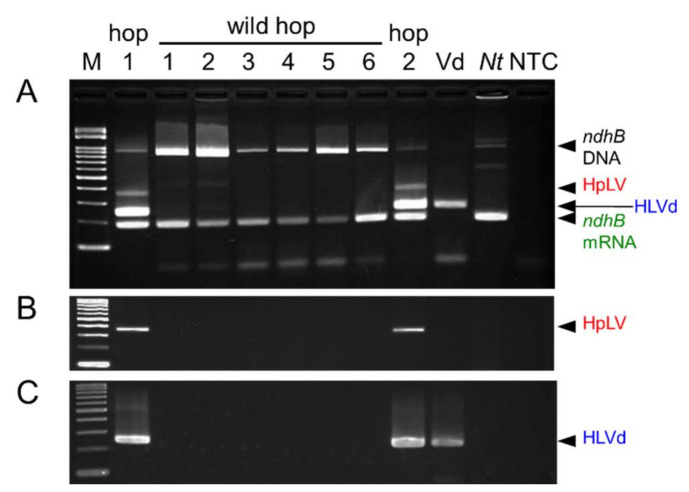
Diagnosis of hop latent virus (HpLV) and hop latent viroid (HLVd) infection in two hop (1 and 2) and six wild hop (1–6) plants. Nucleic acids were extracted by the SPEX-B method and used for triplex RT-PCR detecting HpLV, HLVd, and *ndhB* mRNA (**A**), and simplex RT-PCR detecting HpLV (**B**) or HLVd (**C**). RT-PCR products were electrophoresed in a 3% GenePure 3:1 agarose gel. Lanes, Vd: purified HLVd; *Nt*: purified total RNAs of healthy *N.* *tabacum*; NTC: no template control; M: molecular size marker Gene Ladder 100.

The SPEX method was applied to the practical diagnosis of three crops. Hop latent virus (HpLV, genus *Carlavirus*) and hop latent viroid (HLVd, genus *Cocadviroid*) are widely distributed in hops cultivated worldwide, including in Japan [15,16]. Total nucleic acids of hop (*Humulus lupulus* var. *lupulus*) and wild hop (*H. lupulus* var. *cordifolius*) plants were extracted from leaves sampling early in October by the SPEX-B method. HLVd purified from low molecular weight RNAs of hop leaves infected with both HpLV and HLVd was used as a positive control for the detection of HLVd. In addition, a healthy tobacco leaf was used as a negative control for extraction because a hop plant free of both HpLV and HLVd was not available. The nucleic acids extracted from a healthy tobacco leaf by the SPEX-B method were used as a positive control for the detection of *ndhB* mRNA because it was unknown whether the primer pair of *ndhB* mRNA is applicable to hop and wild hop plants. Moreover, RT-PCR products amplified from the tobacco nucleic acids were used as the size marker of band specific to *ndhB* mRNA. In order to intensify the specific band to *ndhB* mRNA and confirm the disappearance of non-specific *ndhB* DNA band by DNase I treatment before RT-PCR, the tobacco nucleic acids were further purified by DNase I digestion followed by phenol-chloroform extraction. Using total nucleic acids extracted by the SPEX-B method, both HpLV and HLVd were detected in two hop plants, but not from six wild hop plants grown naturally. Meanwhile, the *ndhB* mRNA was detected from both hop and wild hop plants (Figure 4A). The absence of HpLV and HLVd in wild hop plants tested was confirmed also by simplex RT-PCR (Figure 4B,C).

**Figure 5:**
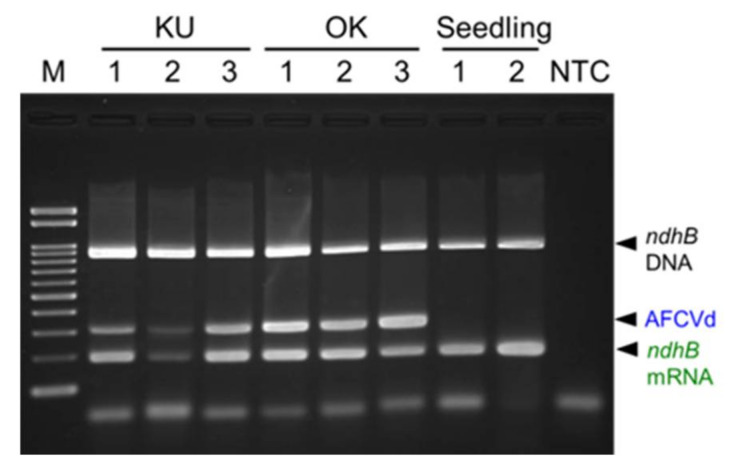
Diagnosis of apple fruit crinkle viroid (AFCVd) infection in Japanese persimmon. Nucleic acids were extracted by the SPEX-B method from pericarps of KU (1–3) and OK (1–3) fruit samples and leaves of two seedlings grown from seeds of ‘Fuyu’ fruits. Duplex RT-PCR products were electrophoresed in a 2% agarose gel. Lanes, NTC: no template control; M: molecular size marker Gene Ladder 100.

Apple fruit crinkle viroid (AFCVd) is a tentative member of the genus *Apscaviroid* and naturally infects apple, hop, and persimmon plants in Japan [17,18,19]. The SPEX-B method was used to detect AFCVd from fruits of Japanese persimmon (*Diospyros kaki*). Total nucleic acids were extracted from frozen pericarps of three fruits for each of persimmon trees, named KU and OK, which were confirmed to be infected with AFCVd in another study. Additionally, total nucleic acids were extracted from fresh leaves of two seedlings, which were previously confirmed as free of AFCVd. AFCVd was successfully detected in persimmon fruit samples KU1, KU3, and OK1–3, and weakly from KU2, but not from the leaves of seedlings. Meanwhile, *ndhB* mRNA could be detected from both pericarps and leaves of Japanese persimmon regardless AFCVd infection although it was weak from KU2 (Figure 5).

**Figure 6:**
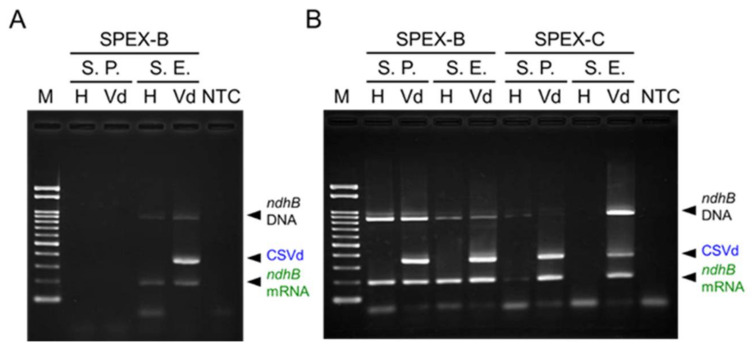
Diagnosis of chrysanthemum stunt viroid (CSVd) infection in two chrysanthemum cultivars, ‘Sei Prince’ (S. P.) and ‘Sei Elza’ (S. E.). Nucleic acids were extracted by the SPEX-B (**A**,**B**) and SPEX-C (**B**) methods, and further purified by differential precipitation with 2-butoxyethanol (**B**). Duplex RT-PCR products were electrophoresed in a 2% agarose gel. Lanes, H: healthy plants; Vd: CSVd-infected plants, NTC: no template control; M: molecular size marker Gene Ladder 100.

In addition, the SPEX method was used to detect CSVd in two cultivars of chrysanthemum (*Chrysanthemum* × *morifolium*). First, the detection of CSVd was attempted using nucleic acids extracted from young leaves by the SPEX-B method; however, no RT-PCR products were amplified from the nucleic acids of ‘Sei Prince’. CSVd and *ndhB* mRNA were detected only from the ‘Sei Elza’, whose leaves were softer and more pliable than that of the ‘Sei Prince’ (Figure 6A). Failure of amplification from the nucleic acids of ‘Sei Prince’ was assumed (from UV absorption spectrophotometry) to be caused by inhibitors such as polysaccharides and phenolic compounds in the impurities. Thereafter, nucleic acids extracted by the SPEX-B or SPEX-C method were further purified by differential precipitation with 2-butoxyethanol (2-BE), as described previously [11,20] with minor modifications. Consequently, CSVd and *ndhB* mRNA could be detected from both ‘Sei Elza’ and ‘Sei Prince’ using the purified nucleic acids by the SPEX-B method followed by 2-BE. Using the purified nucleic acids by the SPEX-C method followed by 2-BE, CSVd and *ndhB* mRNA could also be detected from both ‘Sei Elza’ and ‘Sei Prince’ infected with CSVd; however, *ndhB* mRNA was barely detected from healthy ‘Sei Prince’ and not detected in the healthy ‘Sei Elza’ (Figure 6B). These results indicate that the SPEX-B method stably provide total nucleic acids suitable for the detection of both *ndhB* mRNA and CSVd in chrysanthemum plants compared with the SPEX-C method although further purification by 2-BE is needed.

## 3. Discussion

The previous PEX method [9,10,11] for extracting nucleic acids from plant tissue without mechanical grinding was revised herein as the SPEX method. Besides viroid RNA detection previously reported [11], the method using PEX was demonstrated to be useful for extracting viral single-stranded RNA genome and plant mRNA too for the first time in this study. The PEX buffer treatment was conducted in the original procedure by Jhingan [9] with one step at 65 °C for 20 min; however, the step for the infiltration of the PEX buffer into plant tissue in a microcentrifuge tube using a centrifuge evaporator was added during the incubation at 65 °C for the total of 20 min in the modified procedure by Williams and Ronald [10]. Although this infiltration step was conducted for 6 min in our previous report [11], the occurrence of cross-contamination in this step was of concern because this step was conducted with cap-opened tubes. Based upon our previous report [11], the infiltration step was considered to be effective, but not essential for the plant cell wall disruption; therefore, we attempted to omit it. Moreover, the PEX buffer treatment was shortened from 20 min to 10 min. Instead, we tested to conduct the PEX buffer treatment by incubation at higher temperature than 65 °C. Comparisons among the standard PEX and three SPEX methods did not provide differences for detecting CSVd in tomatoes, CEVd in ‘Etrog’ citrons, PVY in *Nicotiana* plants, and *ndhB* mRNA in these plants (Figure 1A–D). However, when CMV and *ndhB* mRNA in *N. benthamiana* was simultaneously detected, cDNA amplification of CMV or *ndhB* mRNA was unsuccessful in some cases (Figure 1E and Figure S1, Table 1). Although the exact cause of unsuccessful or poor cDNA amplification is unknown even after comparing the UV-absorbance spectra of extracted nucleic acids, the quantity of nucleic acids extracted using the SPEX-A method by incubation at 65 °C appeared to be lower than that using other methods (Table 1, Figure 2). The SPEX-C method by incubation at 95 °C provided the highest concentration of nucleic acid; despite impurities, cDNA of CMV and *ndhB* mRNA were successfully amplified. The quantity and quality of nucleic acids extracted by the SPEX-B method was similar to that by the standard PEX method (Figure 2); therefore, the incubation at 65 °C for the total of 20 min in combination with infiltration step for the PEX buffer treatment in the standard PEX method was replaced with an incubation at 80 °C for 10 min in the SPEX-B method.

The SPEX-B method was applicable to fresh, −80 °C frozen, and silica gel-dried leaves (Figure 3). In addition to the CSVd and CMV, AFCVd could also be detected from −80 °C frozen (Figure 5) and silica gel-dried persimmon pericarps (data not shown). Preserving sample tissue with silica gel is convenient for sampling in fields, although it takes time to dry completely. As a solution to this, subsequent heat drying may be useful for the complete drying of sample tissue because leaves dried at 65 °C for two days have been shown to be suitable for the detection of four viruses and one viroid [21].

The SPEX-B method has been used in our laboratory as a convenient rapid method in the detection of plant viruses and viroids including (in addition to the above), potato spindle tuber viroid and tomato chlorotic dwarf viroid (genus *Pospiviroid*) in cultivated and wild tomato species, hop stunt viroid (genus *Hostuviroid*) in cucumbers [22,23], CSVd in *Glebionis coronaria* (*Chrysanthemum coronarium*), potato aucuba mosaic virus (genus *Potexvirus*) in *Nicotiana* spp. and tomatoes, and potato virus S (genus *Carlavirus*) in *N. occidentalis* [Hataya, unpublished]. Although the target in this study was the RNA sequence, DNA was also considered to be efficiently extracted judging from the amplification of the genomic *ndhB* DNA fragment. The original or modified PEX method was reported to be applicable for the extraction of plant DNA from many monocotyledons and dicotyledons, such as barley (*Hordeum vulgare*), maize (*Zea mays*), oat (*Avena sativa*), rice (*Oryza sativa*), sorghum (*Sorghum vulgare*), triticale (a wheat/rye hybrid), wheat (*Triticum aestivum* and *T. durum*), alfalfa (*Medicago sativa*), common bean (*Phaseolus vulgaris*), lettuce (*Lactuca sativa*), petunia (*Petunia hybrida*), rapeseed (*Brassica napus*), soybean (*Glycine max*), sunflower (*Helianthus annuus*), and tobacco [9,10]. In addition, nucleic acid extraction using PEX-containing buffer has been reported for cyanobacteria and several other bacteria, archaea [9,24,25,26,27,28], and fungi [29,30]. Based upon these reports, the SPEX method has the potential to be applicable for the detection of bacterial and fungal pathogens in plants.

According to the UV-absorbance spectra, nucleic acids extracted by the SPEX-B method from *N. benthamiana* plants appear to be relatively pure with contamination of few proteins, polysaccharides, and polyphenols (Figure 2). However, the non-grinding SPEX method is considered to reduce the yield of nucleic acids compared to other methods with grinding. Jhingan [9] compared between non-grinding and grinding methods using the same PEX buffer for the yield of nucleic acids extracted from various plant leaves. Compared with the grinding PEX method, the yield of nucleic acids by the non-grinding PEX method was decreased from 4.8-fold in rapeseed to 121.9-fold in barley. The RNA yield purified from total nucleic acids extracted from approximately 50 mg of tobacco leaf by the SPEX-B method was lower than 1 µg (data not shown). According to manufacturer’s data of RNAiso Plus (Takara Bio, Shiga, Japan) that is an AGPC (acid guanidinium thiocyanate-phenol-chloroform) reagent [31] and NucleoSpin RNA Plant Kit using a silica membrane spin column (Macherey-Nagel GmbH & Co. KG, Düren, Germany), the RNA yield using the SPEX method is approximately 50-fold and 24-fold lower than that using two methods, respectively. Additionally, degraded ribosomal RNA bands were observed in the agarose gel when the purified total RNAs extracted using three SPEX methods from *Nicotiana* plants were electrophoresed (data not shown). For the reasons above, the nucleic acid extracts by the SPEX method seems to be unsuitable for analyses requiring a certain amount of RNA with high quality such as Northern blot analysis. However, the yield and quality of nucleic acids extracted by the SPEX-B method was sufficient for RT-PCR judging from the cDNA amplification of internal control mRNA in several plants tested, except chrysanthemum. Impurities in nucleic acid extracts from chrysanthemum leaves sometimes inhibit RT-PCR and result in a false negative for CSVd, as described previously [11,32]. Purification of nucleic acids by differential precipitation with 2-BE followed by HCl treatment and ethanol precipitation improved the efficiency of RT-PCR in detecting CSVd in nucleic acids extracted from chrysanthemum leaves by the standard PEX method [11]. The efficiency of RT-PCR for detecting CSVd and *ndhB* mRNA in nucleic acids extracted from ‘Sei Prince’ by the SPEX-B method was improved solely by the 2-BE differential precipitation, without HCl treatment and ethanol precipitation (Figure 6). The HCl treatment followed by ethanol precipitation has the potential to improve the efficiency of RT-PCR as described previously, although it is time consuming and may cause yield loss [11,32]. When the cDNA of CSVd and *ndhB* mRNA could not be amplified from total RNAs extracted using an AGPC reagent (TRIsure; Bioline Reagents Ltd., London, UK) from chrysanthemum ‘Sei Elza’ leaves, the cDNA amplification was significantly improved by the 2-BE differential precipitation (data not shown).

Nucleic acid extraction from woody plants is usually laborious in comparison to herbaceous plants. However, the amplification of viroid cDNA was successful from the nucleic acids of young soft and pliable citrus leaves and persimmon pericarps after extraction by the SPEX method. Meanwhile, the cDNA amplification of *ndhB* mRNA or an actin gene from the nucleic acids of persimmon leaves extracted by the SPEX method was not stable in repeated examinations, and the failure of amplification was not improved by differential precipitation of nucleic acids with 2-BE followed by HCl treatment and ethanol precipitation. RNA extraction from persimmon leaves is laborious, probably due to polysaccharides and polyphenols (including tannins), and RNA could not be extracted sufficiently by an AGPC method [31] using commercially available reagents such as TRIzol Reagent (Thermo Fisher Scientific) and RNAiso Plus (Takara Bio). Ikegami et al. [33] reported that RNA of sufficient quality and quantity from mature persimmon leaves could not be extracted using a commercially available spin column kit, such as RNeasy Plant Mini kit (Qiagen, Venlo, The Netherlands) and FastPure RNA kit (Takara Bio), but could be extracted using a combination of the FastPure RNA kit and Fruit-mate (Takara Bio), in addition to an SDS+PVP method. Accordingly, further improvement may be necessary to establish a reliable SPEX method for the extraction of nucleic acids from persimmon leaves. In addition, further improvement is needed to apply the SPEX method to the extraction of nucleic acids from many woody plants because nucleic acids could not be extracted sufficiently from apple and grape pericarps by the SPEX-B method.

In conclusion, the SPEX method described in this paper requires no tissue grinding and therefore no tissue homogenizer, is quite straightforward, and may reduce the risk of cross-contamination. Moreover, the use of inexpensive chemical reagents and the avoidance of deleterious substances such as phenol-chloroform are appropriate for the extraction of nucleic acids from a large number of plant tissue samples and facilitate automation. The SPEX method may be applicable in detecting not only viruses and viroids but also other plant pathogens such as phytoplasmas, bacteria and fungi in various plant species.

## 4. Materials and Methods

### 4.1. Plant Virus and Viroid Sources and Plant Growth Conditions

A PVY isolate belonging to necrotic strain [34] and a CMV isolate belonging to subgroup IA (Hataya, unpublished) were rub-inoculated onto tobacco ‘Xanthi nc’ and/or *N. benthamiana* leaves dusted with 600 mesh carborundum using a cotton swab. The inoculated or healthy *Nicotiana* plants were grown under fluorescent light on a shelf placed in a room at a controlled temperature of 24 °C. CSVd [35] was rub-inoculated onto the cotyledons of tomato ‘Newskij’ plants using a finger covered with a disposable latex finger cot. The tomatoes were grown at 24 °C in a controlled environment chamber. Healthy or CSVd-inoculated chrysanthemum, ‘Sei Prince’, and ‘Sei Elza’ plants were kindly provided by Emeritus Professor Teruo Sano (Hirosaki University, Japan) and grown in a greenhouse at a controlled temperature of 25 °C. CEVd [36] was inoculated on the stem of ‘Etrog’ citron plants by slashing with contaminated razor blade. The CEVd-infected ‘Etrog’ citrons have been maintained for more than two decades in a greenhouse at a controlled temperature of 28 °C.

Hop ‘Kirin II’ plants infected naturally with both HpLV [15] and HLVd [16] have been maintained in a field of Hokkaido University. Leaves of wild hop plants grown naturally were collected from the grounds of Hokkaido University. Japanese persimmon fruit samples were collected from persimmon trees named KU and OK grown in private gardens in Japan. Thereafter, the peeled pericarps of fruit samples were preserved at −80 °C and used for the detection of AFCVd. Persimmon ‘Fuyu’ fruits were purchased at a market, and their seeds were collected and sown, and leaves of seedlings were used as negative controls because these seedlings were free of AFCVd.

### 4.2. Simplified Extraction of Nucleic Acids Using PEX Buffer

Our previous procedure [11] based on Williams and Ronald [10] was modified to simplify the process of plant cell wall disruption. Leaf discs were cut out from each plant leaf placed onto a folded facial tissue using a disposable multifunction laboratory spatula (smartSpatula, blue, 7 mm diameter, LevGo, Berkeley, CA, USA) or a commercial plastic straw (6 mm diameter) whose length was cut to 4.0–4.5 cm before use. Approximately 50–100 mg of leaf discs was placed in a 2.0 mL centrifuge tube and washed with 500 µL of 70% ethanol using a vortex mixer. After low-speed spin-down, 70% ethanol was discarded, and the leaf discs were submerged in 500 µL of PEX buffer (6.25 mM PEX (Tokyo Chemical Industry, Tokyo, Japan); 100 mM Tris-HCl, pH 7.5; 700 mM NaCl; 10 mM EDTA, pH 8.0) and were incubated for 10 min in a dry bath. The incubation conditions were compared at 65 °C (SPEX-A), 80 °C (SPEX-B), and 95 °C (SPEX-C). The standard PEX method consisted of an incubation at 65 °C for 5 min followed by infiltration at 65 °C for 6 min using a vacuum evaporator and incubation again at 65 °C for 15 min [10]. Subsequently, 400 µL of PEX buffer was retrieved into a 1.5 mL centrifuge tube after vortex mixing for 10 s and low-speed spin-down. An equal volume of 2-propanol was added to the tube and mixed by vigorous vortexing. Total nucleic acids were collected by centrifugation at 16,000× *g* for 5 min at 4 °C, rinsed with 500 µL of 70% ethanol, air-dried, and dissolved in 20 µL of ultrapure water by vigorous vortexing. If an undissolved pellet appeared after low-speed spin-down, the supernatant was used for RT-PCR. Depending on the amount of visible pellet after 2-propanol precipitation, 1–2 µL of the dissolved nucleic acids was used for RT-PCR.

### 4.3. Differential Precipitation of Nucleic Acids with 2-Butoxyethanol

In the case of chrysanthemum plants, total nucleic acids extracted from leaves by the SPEX method described above were further purified by differential precipitation with 2-BE (FUJIFILM Wako Pure Chemical, Osaka, Japan) as described previously [11,20] with minor modifications. Total nucleic acids extracted by the SPEX method were dissolved in 50 µL of ultrapure water. Subsequently, an equal volume of 2× TBEN buffer (100 mM Tris-HCl, pH 7.6; 50 mM boric acid; 2.5 mM EDTA, pH 8.0; 200 mM NaCl) was added to the nucleic acid solution and mixed well. Thereafter, 0.4 volumes (40 µL) of 2-BE were added to the nucleic acid solution and vigorously mixed by vortexing. After standing on ice for 30 min and centrifuging at 16,000× *g* for 5 min at 4 °C, the gel-like polysaccharide pellet was discarded, and the supernatant was transferred into a new tube. Afterwards, 0.6 volumes (60 µL) of 2-BE were added to the solution and vigorously mixed by vortexing. After standing on ice for 30 min and centrifuging at 16,000× *g* for 5 min at 4 °C, the supernatant containing phenolic compounds was discarded. The pellet was rinsed with 400 µL of 70% ethanol, air-dried, and dissolved in 10 µL of ultrapure water by vigorous vortexing. Two microliters of the purified nucleic acid solution was used for RT-PCR.

### 4.4. RT-PCR

The cDNA was synthesized in a 10 µL reaction mixture containing 0.5 µL (50 U) of reverse transcriptase ReverTra Ace (Toyobo, Osaka, Japan), 1× buffer (50 mM Tris-HCl, pH 8.3; 75 mM KCl; 3 mM MgCl_2_; 10 mM DTT), 1 mM each of dNTP, and 1.25 µM random hexamer (Takara Bio) with 1–2 µL of the extracted nucleic acid solution. The reaction mixture was incubated at 30 °C for 10 min and subsequently at 42 °C for 20 min. After the reaction, reverse transcriptase was inactivated by heating at 85 °C for 5 min.

PCR was carried out in a 20 µL reaction mixture containing 0.4 µL (1 U) of Go-to DNA polymerase (Nippon Gene, Toyama, Japan), 1× buffer (supplied by Nippon Gene), 0.2 mM each of dNTP, 0.2 µM each of internal control primer set AtropaNad2.1a and AtropaNad2.2b (except simplex PCR), one or two (triplex PCR for HpLV and HLVd) sets of 0.2 µM of each target primer (Table S1, [11–13,15,16,35,37]) with 1 µL of cDNA products. The cDNA was amplified by an initial denaturation at 95 °C for 2 min, 35 cycles of denaturation at 95 °C for 20 s, annealing at 50–61°C depending on the primer set (Table S1) for 20 s, extension at 72 °C for 10 s, and a final extension at 72 °C for 3 min. Alternatively, PCR was carried out using Hot Start *Taq* DNA polymerase (New England Biolabs, Ipswich, MA, USA) to compare Go-to DNA polymerase and Hot Start *Taq* DNA polymerase for the amplification of cDNA of CSVd and *ndhB* mRNA, and *ndhB* DNA. The cDNA was amplified in a 20 µL reaction mixture containing 0.1 µL (0.5 U) of Hot Start *Taq* DNA polymerase (New England Biolabs), 1× buffer (supplied by New England Biolabs), 0.2 mM each of dNTP, 0.2 µM each of internal control primer set AtropaNad2.1a and AtropaNad2.2b, 0.2 µM each of target primer set CSV-1P and CSV-1M (Table S1) with 1 µL of cDNA products. The PCR thermal cycling conditions were as follows: an initial denaturation at 95 °C for 3 min; 35 cycles of denaturation at 95 °C for 20 s, annealing at 55 °C for 30 s, extension at 68 °C for 30 s; and a final extension at 72 °C for 3 min.

Half of the RT-PCR products (10 µL) were electrophoresed in a 2% agarose or 3% GenePure 3:1 agarose (ISC BioExpress, Kaysville, UT, USA) gel using 0.5× TAE (20 mM Tris-acetate, 0.5 mM EDTA) running buffer, and visualized under UV light (312 nm) after staining with ethidium bromide (0.5 µg/mL). Gene Ladder 100 (Nippon Gene) was used as a molecular size marker.

### 4.5. Quantity and Quality Evaluation of Extracted Nucleic Acids

The quantities and qualities of nucleic acids extracted from leaves of *N. benthamiana* plants by the standard PEX and three SPEX methods were evaluated using a NanoDrop™ 1000 spectrophotometer (Thermo Fisher Scientific, Waltham, MA, USA). The third, fourth, and fifth nucleic acid extracts of *N. benthamiana* infected with CMV, which were used for the simultaneous detection of CMV and *ndhB* mRNA by RT-PCR (Table 1) and stored at −80 °C, were used for the evaluation. The absorbance value was measured three times per nucleic acid extract and the average was calculated.

## Figures and Tables

**Table 1:** Comparison of four methods for detecting cucumber mosaic virus.

Exp.	Target	Standard PEX	SPEX-A	SPEX-B	SPEX-C
1st (10 dpi)	CMV	+++	(+)	+++	+++
	*ndhB* mRNA	+	−	++	+
2nd (14 dpi)	CMV	+	+++	+++	+++
	*ndhB* mRNA	−	++	++	++
3rd (10 dpi)	CMV	+++	+++	+++	+++
	*ndhB* mRNA	+	+	+	+
4th (14 dpi)	CMV	+	+	+++	+++
	*ndhB* mRNA	+	−	++	++
5th (14 dpi)	CMV	+++	++	+++	+++
	*ndhB* mRNA	++	+	++	+

Cucumber mosaic virus (CMV) and *ndhB* mRNAs were simultaneously detected from nucleic acid extracts of *N. benthamiana* 10 days post-inoculation (dpi) or 14 dpi. The presence and absence of amplified products by agarose gel analysis is shown with symbols “+” and “−”, respectively. The band intensity is shown as follows: +++ > ++ > + > (+).

## Data Availability

Not applicable.

## Supplementary Materials

The following are available online at https://www.mdpi.com/article/10.3390/plants10122683/s1, Figure S1: Comparison of incubation conditions in the SPEX method for detecting cucumber mosaic virus (CMV) in *N*. benthamiana, Table S1: List of primers used in PCR.
