# Supplementary material for: An Improved Method for the Extraction of Nucleic Acids from Plant Tissue without Grinding to Detect Plant Viruses and Viroids
Source: Plants (Basel). 2021 Dec 6;10(12):2683. doi: 10.3390/plants10122683 (PMC8708111; doi:10.3390/plants10122683)
Supplement: Supplementary file 1 [file plants-10-02683-s001.zip › plants-1482166-supplementary.pdf]

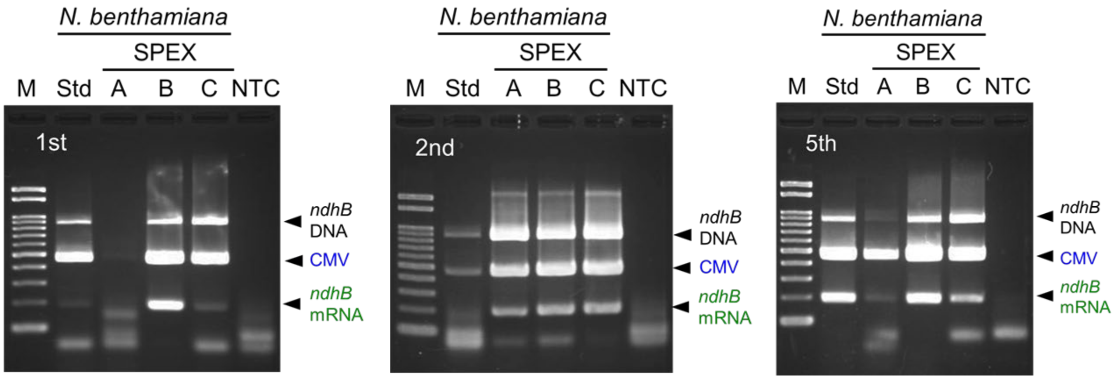

Figure S1. Comparison of incubation conditions in the SPEX method for detecting cucumber mosaic virus (CMV) in *N. benthamiana*. CMV cDNA was detected simultaneously with the cDNA of *ndhB* mRNA by RT-PCR using Go-to DNA polymerase from nucleic acids extracted from leaves using the standard PEX (lane Std), and SPEX-A, SPEX-B, and SPEX-C (lanes A–C) methods. Duplex RT-PCR products were electrophoresed in a 2% agarose gel. Lanes, NTC: no template control; M: molecular size marker Gene Ladder 100.

Table S1. List of primers used in PCR

Target	Primer name	Primer sequence 5′–3′	Annealing temp. (°C)	Size of PCR product (bp)	Ref.
chrysanthemum stunt viroid (CSVd)	CSV-1P<br>CSV-1M	CTTAGGACCCCACTCCTGCG<br>CCGCGATCTCGTCGGACTTC	61	348	[11,35]
citrus exocortis viroid (CEVd)	PCEV-1P<br>PCEV-1M	GCTCCACATCCGATCGTC<br>TGGACGCCAGTGATCCGC	50	332	[11]
potato virus Y (PVY) *coat protein*	PVYCP6P<br>PVYCP6M	CGTCCAAAATGAGAATGCC<br>TCTTGTGTACTGATGCCAC	55	577	[37]
cucumber mosaic virus (CMV) *2a*	CM2a-1P<br>CM2a-1M	TTCCAGAGATGCCTTCGAGAACG<br>TCCATCACCTTAGCTTCCATGTTG	55	470	This study
apple fruit crinkle viroid (AFCVd)	AFCV-5P<br>AFCV-5M	GCCCTGGGCTCCAACTAGTGG<br>ACTGGTTGGGACCGCTGGGAC	55	308	This study
hop latent viroid (HLVd)	HLVd-1P<br>HLVd-1M	GGATACAACTCTTGAGCGCC<br>TAGTTTCCAACTCCGGCTGG	50	250	[16]
hop latent virus (HpLV) *replicase*	HLV-5P<br>HLV-9M	GCAAAAGCAGCGCAGAGTATAG<br>TCGCCTGAGAAATGCATTATAGC	50	359	[15]
ndhB[a] mRNA	AtropaNad2.1a<br>AtropaNad2.2b	GGACTCCTGACGTATACGAAGGATC<br>AGCAATGAGATTCCCCAATATCAT	50–61	188	[12,13]

[a] NADH dehydrogenase subunit 2 gene
